# Impact of a clinical decision support tool on prediction of progression in early-stage dementia: a prospective validation study

**DOI:** 10.1186/s13195-019-0482-3

**Published:** 2019-03-20

**Authors:** Marie Bruun, Kristian S. Frederiksen, Hanneke F. M. Rhodius-Meester, Marta Baroni, Le Gjerum, Juha Koikkalainen, Timo Urhemaa, Antti Tolonen, Mark van Gils, Daniel Rueckert, Nadia Dyremose, Birgitte B. Andersen, Afina W. Lemstra, Merja Hallikainen, Sudhir Kurl, Sanna-Kaisa Herukka, Anne M. Remes, Gunhild Waldemar, Hilkka Soininen, Patrizia Mecocci, Wiesje M. van der Flier, Jyrki Lötjönen, Steen G. Hasselbalch

**Affiliations:** 1 Danish Dementia Research Centre, Neuroscience Centre, Department of Neurology, Rigshospitalet, University of Copenhagen, Copenhagen University Hospital, Blegdamsvej 9, 2100 Copenhagen, Denmark; 2 Alzheimer Center Amsterdam, Department of Neurology, Amsterdam Neuroscience, Vrije Universiteit Amsterdam, Amsterdam UMC, Amsterdam, The Netherlands; 3 Institute of Gerontology and Geriatrics, University of Perugia, Perugia, Italy; 4 Combinostics Ltd., Tampere, Finland; 5 VTT Technical Research Centre of Finland Ltd, Tampere, Finland; 6 Department of Computing, Imperial College London, London, UK; 7 Neurology, Institute of Clinical Medicine, University of Eastern Finland, Kuopio, Finland; 8 Medical Research Center, Oulu University Hospital, Oulu, Finland; 9 Neurology, Neuro Center, Kuopio University Hospital, Kuopio, Finland; 10 Neurology, Unit of Clinical Neuroscience, University of Oulu, Oulu, Finland

**Keywords:** Dementia, Alzheimer’s disease, Conversion, Progression, Mild cognitive impairment, Subjective cognitive decline, CDSS, Computer-assisted

## Abstract

**Background:**

In clinical practice, it is often difficult to predict which patients with cognitive complaints or impairment will progress or remain stable. We assessed the impact of using a clinical decision support system, the PredictND tool, to predict progression in patients with subjective cognitive decline (SCD) and mild cognitive impairment (MCI) in memory clinics.

**Methods:**

In this prospective multicenter study, we included 429 patients with SCD (*n* = 230) and MCI (*n* = 199) (female 54%, age 67 ± 9, MMSE 28 ± 2) and followed them for at least 12 months. Based on all available patient baseline data (demographics, cognitive tests, cerebrospinal fluid biomarkers, and MRI), the PredictND tool provides a comprehensive overview of the data and a classification defining the likelihood of progression. At baseline, a clinician defined an expected follow-up diagnosis and estimated the level of confidence in their prediction using a visual analogue scale (VAS, 0–100%), first without and subsequently with the PredictND tool. As outcome measure, we defined clinical progression as progression from SCD to MCI or dementia, and from MCI to dementia. Correspondence between the expected and the actual clinical progression at follow-up defined the prognostic accuracy.

**Results:**

After a mean follow-up time of 1.7 ± 0.4 years, 21 (9%) SCD and 63 (32%) MCI had progressed. When using the PredictND tool, the overall prognostic accuracy was unaffected (0.4%, 95%CI − 3.0%; + 3.9%; *p* = 0.79). However, restricting the analysis to patients with more certain classifications (*n* = 203), we found an increase of 3% in the accuracy (95%CI − 0.6%; + 6.5%; *p* = 0.11). Furthermore, for this subgroup, the tool alone showed a statistically significant increase in the prognostic accuracy compared to the evaluation without tool (6.4%, 95%CI 2.1%; 10.7%; *p* = 0.004). Specifically, the negative predictive value was high. Moreover, confidence in the prediction increased significantly (∆VAS = 4%, *p* < .0001).

**Conclusions:**

Adding the PredictND tool to the clinical evaluation increased clinicians’ confidence. Furthermore, the results indicate that the tool has the potential to improve prediction of progression for patients with more certain classifications.

**Electronic supplementary material:**

The online version of this article (10.1186/s13195-019-0482-3) contains supplementary material, which is available to authorized users.

## Background

A large proportion of patients referred to memory clinics present with mild cognitive impairment (MCI) or subjective cognitive decline (SCD). Patients with SCD show no detectable deficit in cognition, but have an increased risk of progression and of developing Alzheimer’s disease (AD) dementia [1–3], whereas the term MCI refers to patients already showing objective signs of cognitive dysfunction [4]. The estimated annual conversion rate for patients with MCI is 5–10% [5]. However, not all MCI patients will progress and studies have shown that over a period of 10 years, less than 50% will have developed dementia [4, 5]. For clinicians, it is often difficult to identify which patients will remain stable or progress [2, 5]. However, individualized risk management and early detection of individuals with a progressive condition are important for providing optimal counseling, care, and treatment [6, 7].

Pathophysiological abnormalities are known to precede clinical symptoms of AD [3, 8]. Positive diagnostic tests, such as decreased amyloid-β 1–42 (Aβ42) and elevated tau proteins in cerebrospinal fluid (CSF), *APOE* genotype, and atrophy on *magnetic resonance imaging* (MRI), are associated with an increased risk of progression in patients with MCI and SCD [2, 3, 9–13]. Nevertheless, clinicians struggle to translate this information into clinical decision-making and accurately predict whether the individual patient will progress or not. The latest diagnostic criteria acknowledge the role of biomarkers in predementia stages of AD [4]. However, guidance regarding the prognostic value of the biomarkers and how to combine them optimally is still inadequate [10, 14]. Therefore, clinicians may experience ethical dilemmas when applying biomarkers, as well as communicating biomarker results, without knowing the actual prognostic consequences for the individual patient [15]. Modern clinical decision support systems provide a potential solution as they can assess probabilities of individuals rather than provide only statistical differences at a group level. Furthermore, the decision support systems can assist clinicians in clinical practice by providing an objective and consistent comparison of multivariate and multimodal data [16].

Most classifiers use mainly MRI or combined MRI and CSF biomarkers [17], but classifiers including more clinical variables into the progression model have also been introduced [18, 19]. The disease state index (DSI) classifier integrates all available baseline data (demographics, cognitive tests, CSF biomarkers, and MRI visual and computed ratings) and provides an index defining the likelihood of progression for the individual patient [19]. Previously, using retrospective data, we have shown that this classifier could discriminate between stable and progressive conditions for MCI, as well as SCD patients [20–25]. In this study, we used a decision support tool, the PredictND tool, which apart from the likelihood index of the DSI classifier, also gives the clinician an additional comprehensive visual overview and easily interpretable analysis of all data [19, 26]. In general, decision support tools predicting progression of MCI have predominately been tested on retrospective AD cohorts [17, 27, 28]. However, to establish which impact decision support tools may have on the complex decision-making in daily clinical practice, evaluation in clinical settings is needed.

In this prospective multicenter study, we assessed the clinical impact of the PredictND tool on the clinician’s prediction of clinical progression for patients with SCD and MCI in a mixed memory clinic cohort by comparing the prognostic evaluation without and with the PredictND tool. Our hypothesis was that aid from the PredictND tool would increase the number of correct predictions and the clinicians’ confidence in the predictions.

## Methods

### Study design and participants

**Fig. 1:**
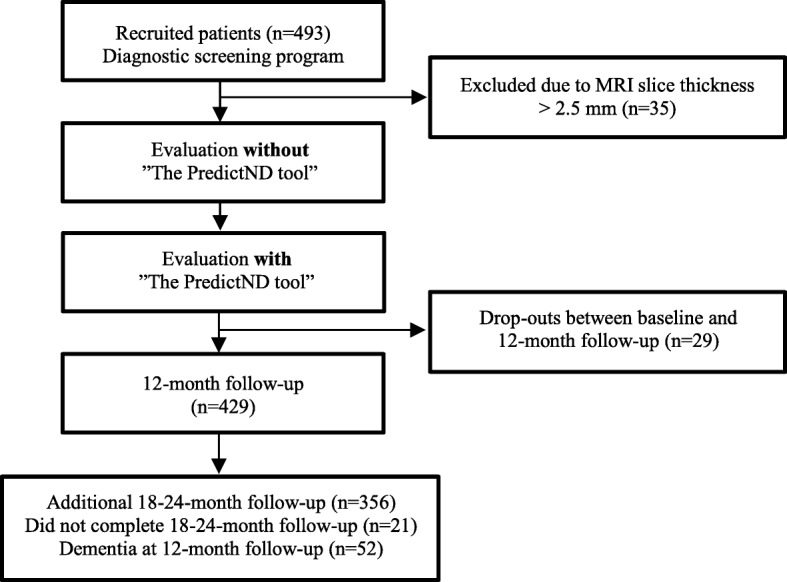
Flow diagram of the study

We recruited patients from four European memory clinics as part of the PredictND project described in detail elsewhere [29]. The patients were enrolled consecutively from March 2015 to June 2016 and followed for a minimum of 1 year. The included patients were diagnosed with either SCD or MCI, had a baseline Mini-Mental State Examination (MMSE) ≥ 18, Clinical Dementia Rating (CDR) ≤ 1.0, and a T1-weighted MRI at or above 1.5 Tesla within the last 6 months with a decent image resolution (slice thickness < 2.5 mm). The exclusion criteria were a major psychiatric disorder, excessive alcohol intake, or substance abuse within the last 2 years, and other brain disorders, which could explain the cognitive problems. We recruited 493 patients, of whom 29 dropped out before the follow-up visit and 35 were excluded, leaving a total number of 429 patients with SCD (*n* = 230) and MCI (*n* = 199) available for analysis (Fig. 1).

All patients underwent a standard multidisciplinary diagnostic screening program including medical history, neurological and physical examination, cognitive testing, blood screening, and an MRI scan. When considered clinically relevant, the assessment program was supplemented with additional tests, such as CSF biomarkers, 18F-fluorodeoxyglucose positron emissions tomography (18F-FDG-PET), or amyloid PET. Patients with cognitive complaints were diagnosed with SCD if the criteria for MCI, dementia, or other disorders are known to cause cognitive problems were not met. MCI was diagnosed according to the National Institute on Aging-Alzheimer’s Association (NIA-AA) criteria [4], whereas AD dementia was diagnosed according to NIA-AA criteria for AD dementia [30], and other types of dementia were diagnosed using established clinical criteria [31–33].

The study was approved by the local Medical Ethical Committee in all four centers. All patients provided written informed consent for their data to be used for research purposes.

### Assessment of impact

The study was designed to mimic clinical practice. Therefore, a single clinician performed the diagnostic evaluation according to usual practice based on a clinical impression, all available information from medical history, clinical investigations, paraclinical results, and scans. For all patients, the clinician stated the current diagnosis (SCD/MCI), the expected follow-up diagnosis (SCD/MCI/dementia), and their level of confidence in the estimated prediction as high, moderate, or low, and on a visual analogue scale (VAS) from 0 to 100%. The same (single) clinician re-evaluated the patients using the same available information and test results but also assisted by the PredictND tool. Again, the expected follow-up diagnosis and the clinician’s level of confidence in the prediction were recorded. All evaluations were performed as close to baseline as possible. For the clinician to recall the clinical impression of the patient, we aimed to perform the re-evaluations within 30 days from the initial evaluation without tool (median = 28, IQR 0–123). The evaluations were performed by clinicians (*n* = 8), who had all received basic training on how to use the PredictND tool.

All patients had a clinical follow-up visit after 12 months. If the patient had progressed to dementia, no further follow-up was conducted, whereas patients with SCD or MCI were followed for additional 6–12 months (Fig. 1). At each follow-up visit, including at least MMSE, CDR, and a clinical interview, a follow-up reference diagnosis was determined by a clinician who had no knowledge of the baseline prediction. In a subset of patients (*n* = 21, 5%), a clinical follow-up visit was not possible, and the diagnostic evaluation was based on a telephone interview. As outcome measure, we defined clinical progression as progression from SCD to MCI or dementia, and from MCI to dementia as diagnosed at follow-up. The expected follow-up diagnosis estimated at baseline compared to the baseline diagnosis defined the clinicians’ prediction of progression as either stable or progression. Correspondence between the prediction of progression and the actual clinical progression defined the prognostic accuracy.

### The PredictND tool

The PredictND tool is a clinical decision support tool designed to assist clinicians in differential diagnosis of dementia and to predict whether the condition will progress or remain stable [19, 26, 34, 35]. The tool uses a data-driven classifier, which provides a scalar disease state index (DSI) value between zero and one. In this study, DSI indicates the likelihood of progression [19]. The model was developed based on training data from patients with established diagnoses of AD and controls. A DSI value close to zero indicates that a given patient resembles controls in the database and is more likely to remain stable, whereas a DSI value close to one indicates a high likelihood of progression to dementia due to AD. Thus, patients with a low or high DSI value are typically more likely correctly classified than patients with medium DSI values. The DSI classifier is described in detail elsewhere [19, 26].

The DSI analysis can handle different types of variables, such as demographic information, cognitive tests, CSF biomarkers, APOE genotyping, and MRI visual and computed ratings, and tolerate missing data [26, 35–37]. The DSI is computed using the following methods: (1) Each variable of the patient’s data is compared with the training data using a fitness function defined as *f*(*x*) = FN(*x*)/(FN(*x*) + FP(*x*), where FN(*x*) is the false negative errors and FP(*x*) the false positive errors in the training data, when using *x* as the classification cutoff value. (2) A relevance function for each variable is defined as relevance = sensitivity + specificity – 1. (3) Finally, the fitness values weighted according to their relevance produce a composite DSI defined through DSI = ∑ (relevance × fitness)/∑ relevance; see a detailed description in [19, 26].

**Fig. 2:**
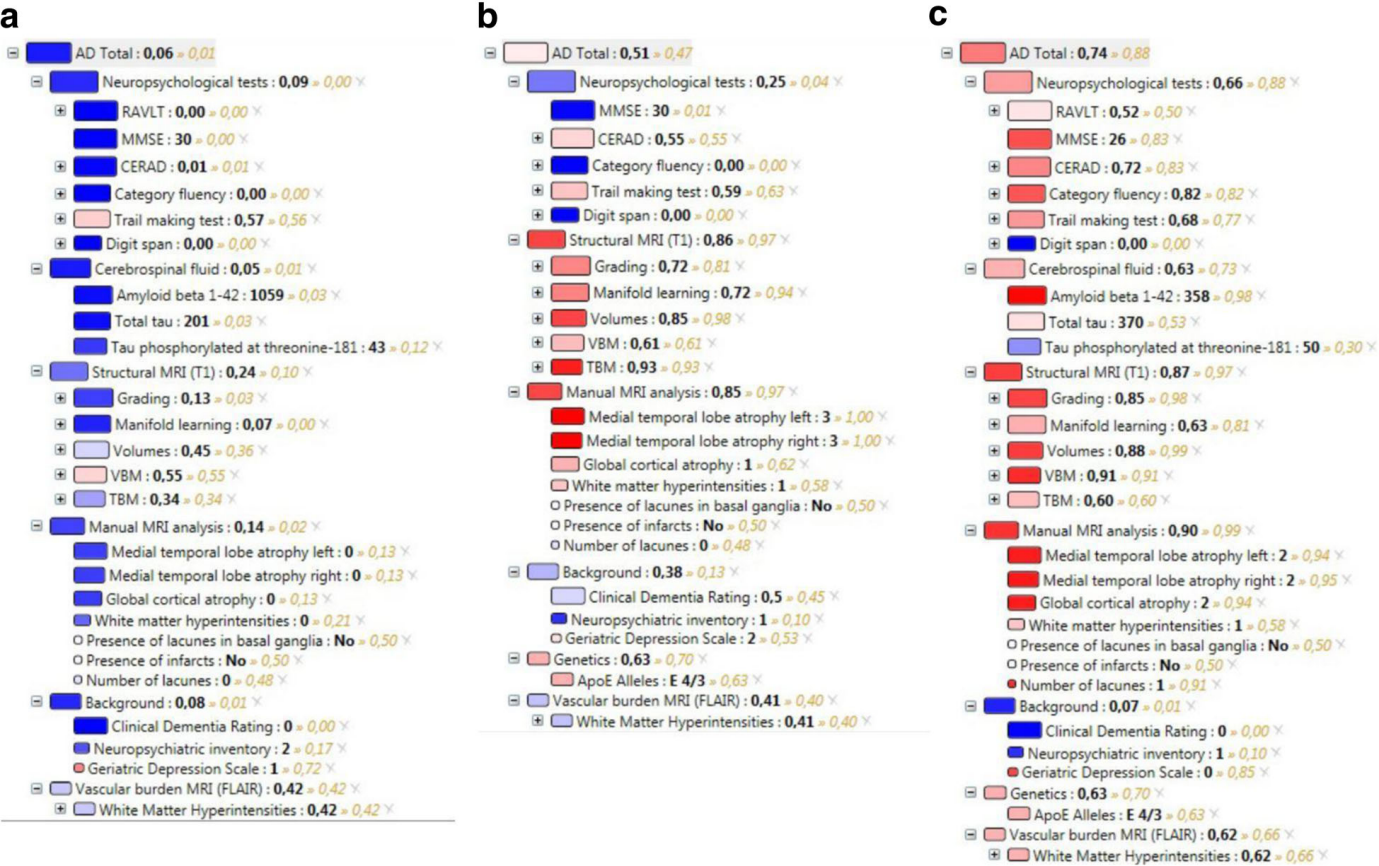
Disease state fingerprints from the PredictND tool. **a** A 46-year-old male with a family history of early-onset dementia and 1 year of word-finding problems, loss of initiative, and subjective complaints of affected memory and sleep. Aβ42, 1059 ng/L; total tau, 201 ng/L; and P-tau, 43 ng/L. Diagnosed with SCD at baseline. The DSI value (0.06) predicted a stable condition and increased the clinician’s confidence from a VAS score of 55% to 80% in the prediction of stable SCD. After the 18-month follow-up, the diagnosis was still stable SCD. **b** A 76-year-old female with mild forgetfulness for words and names during the last 2 years. Normal daily function. MMSE, 30; CERAD learning, 21/30; and CERAD recall, 1/10. Diagnosed with MCI at baseline. The DSI value (0.51) did not indicate a clear stable or progressive condition. The clinician without tool predicted the follow-up diagnosis to be stable MCI, whereas the clinician with tool predicted the patient to progress to AD dementia. The diagnosis at 24-month follow-up was MCI, but after the end of the project at a 3-year follow-up visit, the patient was diagnosed with AD dementia. **c** A 74-year-old male with memory problems for events and names, loss of initiative, and orientation. Aβ42, 358 ng/L; total tau, 370 ng/L; and P-tau, 50 ng/L. Diagnosed with amnestic MCI at baseline. The DSI value (0.78) predicted progression and increased the clinician’s confidence in the prediction of progression to dementia by 30% on the VAS scale. After the 12-month follow-up, the patient had progressed to AD dementia

A graphical tree structure, called the disease state fingerprint (DSF), visualizes how each test or biomarker contributes to the DSI classification [19, 26]. The fit of the patient data to the training data is displayed on a color scale (blue shades indicating controls and red shades AD) and the weight by which each data point contributes to the prediction with the sizes of the boxes. This makes interpretation of the result easier for the clinician. Figure 2 shows examples of DSF visualizations from the PredictND tool.

### Clinical data

Cognition was assessed using a standardized cognitive test battery, as presented in Table 2 and described in [29]. Lumbar puncture was performed on clinical indication (*n* = 145), and Aβ42, total tau, and tau phosphorylated at threonine 181 were measured with commercially available ELISA (Innotest, Fujirebio Europe, Ghent, Belgium). MRI scans were acquired on either 1.5T or 3T scanners, including a T1-weighted gradient echo sequence and a fast fluid-attenuated inversion recovery (FLAIR) sequence. Visual MRI rating was performed using the following: global cortical atrophy (GCA), medial temporal lobe atrophy (MTA), and Fazekas scale for white matter hyperintensities (WMH) [38–40]. Additionally, the PredictND tool extracted imaging biomarkers from the MRI scans using the following automatic quantification methods: hippocampus segmentation measuring volume, tensor-based morphometry analyzing changes in the local volume, voxel-based morphometry analyzing changes in the local gray matter concentration, manifold learning finding low-dimensional representation of high-dimensional data, region-of-interest (ROI) grading comparing similarity of intensities within ROIs and vascular burden combining volume of white matter hyperintensities (WMH), volume of cortical infarcts, and volume of lacunar infarcts. All methods are described in [34].

### Statistical analysis

Differences in baseline characteristics between groups were assessed using independent *t* test and chi-square test, where appropriate.

First, we assessed the effect of the PredictND tool on the clinicians’ prediction of progression. Baseline predictions were defined as either concordant or discordant according to whether the prediction changed or remained unchanged after using the PredictND tool. Moreover, the predictions were defined as correct or incorrect based on whether or not they corresponded to the clinical progression outcome at follow-up. The impact of the PredictND tool on the prognostic accuracy was calculated based on the difference between paired proportions and tested using McNemar’s test. Subsequently, we repeated the analysis including only progression to MCI or dementia due to AD (excluding other types of dementias). Further, we restricted the analysis to a subset of the cohort with more certain DSI classifications; either high probability of progression (DSI ≥ 0.8) or low probability of progression (DSI ≤ 0.2). For evaluation of performance, we used the following metrics: sensitivity, specificity, negative predictive value (NPV), positive predictive value (PPV), accuracy, and balanced accuracy (defined as the average of sensitivity and specificity). In the DSI classification, the cutoff value for progression was defined as a DSI ≥ 0.5.

Finally, paired-sample *t* tests were used to assess change in the level of confidence in the prediction (VAS 0–100%) after applying the PredictND tool to the evaluation. The level of significance was set at *p* value < 0.05 (two-tailed). SAS enterprise guide 7.1 was used for the analyses (SAS Institute, Inc., Cary, NC, USA).

## Results

### Participants

Baseline characteristics are presented in Table 1. After a mean of 1.7 ± 0.4 years, 21 (9%) of the patients with SCD had progressed to either MCI (*n* = 16), AD (*n* = 3), or non-AD dementia (*n* = 2, mixed dementia). Of the patients with MCI, 63 (32%) progressed to AD dementia (*n* = 41) or non-AD dementia (*n* = 22, 4 frontotemporal dementia, 3 dementia with Lewy bodies, 4 vascular dementia, 5 mixed dementia, 6 other types of dementia). Baseline characteristics according to the outcome at follow-up are presented in Table 2 (stratified by SCD and MCI in Additional file 1: Table S1).

**Table 1:** Baseline characteristics

Characteristic	SCD *n* = 230	MCI *n* = 199	*p* value
Female, *n* (%)	150 (65)	80 (40)	< .0001
Age, years	64 (9)	70 (9)	< .0001
Duration of symptoms, years	3 (4)	2 (3)	0.009
MMSE	29 (1)	27 (3)	< .0001
Follow-up time, years	1.9 (0.3)	1.6 (0.5)	< .0001
Progressed, *n* (%)	21 (9)	63 (32)	< .0001
Outcome: MCI/AD/non-AD, *n*	16/3/2	−/41/22	NA

Differences between groups were assessed using independent *t* test and chi-square test. Data are presented as mean ± SD or number (%)

*Abbreviations: SCD* subjective cognitive decline, *MCI* mild cognitive impairment, *MMSE* Mini-Mental State Examination

**Table 2:** Baseline characteristics according to the outcome at follow-up

Characteristic	*n*	Stable	Progressed	*p* value
		*n* = 345	*n* = 84	
Demographics				
Female, *n* (%)	429	191 (55)	39 (46)	< .0001
Age, years	429	65 ± 9	72 ± 8	< .0001
Duration of symptoms, years	380	3 ± 3	3 ± 3	0.36
MCI/AD/non-AD, *n*		–	16/44/24	NA
CDR, *n* (0.0/0.5/1.0)	424	198/136/6	11/52/4	NA
APOE status				
APOE e4 carrier, *n* (%)	146	50 (14)	14 (17)	0.12
Cognitive tests				
MMSE	427	28 ± 2	26 ± 3	< .0001
Memory—learning	420	42 ± 11	31 ± 10	< .0001
Memory—recall	420	9 ± 4	4 ± 3	< .0001
TMT-A, seconds	422	42 ± 19	58 ± 30	< .0001
TMT-B, seconds	402	102 ± 60	164 ± 82	< .0001
Fluency—animal	407	23 ± 7	18 ± 6	< .0001
Fluency—letter	377	14 ± 5	12 ± 5	0.006
Clock-drawing	394	3 ± 1	2 ± 1	< .0001
CSF				
Aβ42, pg/ml	145	933 ± 285	748 ± 338	0.002
P-tau, pg/ml	145	53 ± 24	63 ± 33	0.05
Total tau, pg/ml	145	348 ± 197	445 ± 318	0.03
MRI—visual scores				
GCA (median, Q1–Q3)	418	0.7 ± 0.7 (1, 0–1)	1.2 ± 0.8 (1, 1–2)	< .0001
MTA, right (median, Q1–Q3)	398	0.6 ± 0.8 (0, 0–1)	1.4 ± 1.0 (1, 1–2)	< .0001
MTA, left (median, Q1–Q3)	398	0.6 ± 0.8 (0, 0–1)	1.6 ± 1.0 (1, 1–2)	< .0001
Fazekas score (median, Q1–Q3)	420	0.8 ± 0.8 (1, 0–1)	1.1 ± 0.8 (1, 1–2)	0.009

Differences between groups were assessed using independent *t* test and chi-square test. Data are presented as mean ± SD unless otherwise specified

*Abbreviations: CDR* clinical dementia rating (global score, range 0–3); *MMSE* Mini-Mental State Examination; *Memory* Rey Auditory Verbal Learning Test (RAVLT) values, using *z*-scoring for those with only the Consortium to Establish a Registry for Alzheimer’s Disease (CERAD) word list memory test; *TMT* Trail Making Test; *CSF* cerebrospinal fluid; *Aβ42* amyloid beta 1–42; *P-tau* tau phosphorylated at threonine 181; *MRI* magnetic resonance imaging; *GCA* global cortical atrophy; *MTA* medial temporal lobe atrophy

### Prediction of progression without and with the PredictND tool

**Fig. 3:**
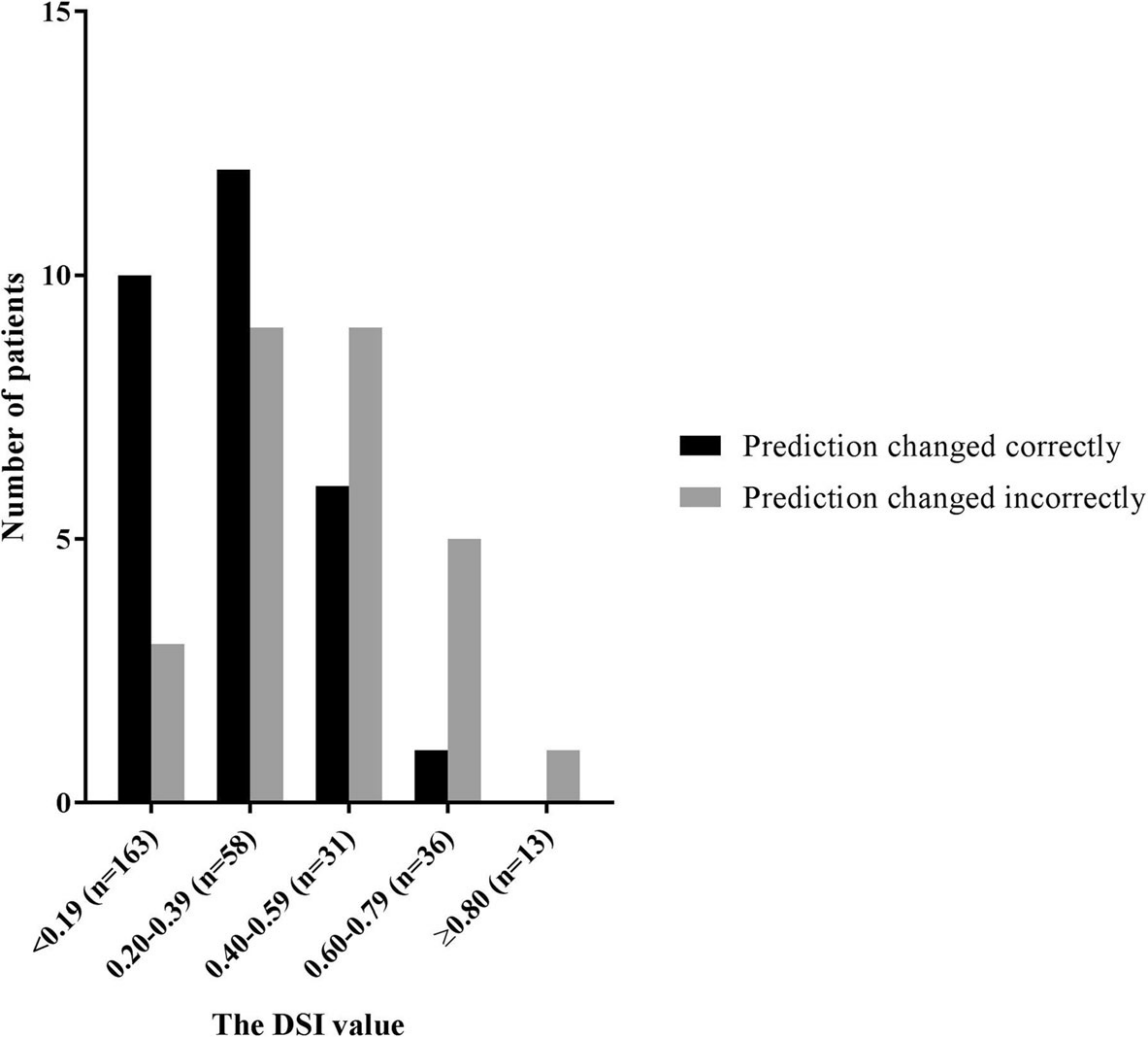
The number of changed predictions after application of the PredictND tool, stratified by DSI values. DSI disease state index

In 56 (13%) patients, the clinician changed the prediction of progression when using the tool. The prediction changed correctly in 29 (7%) patients and incorrectly in 27 (6%) patients compared to the follow-up diagnosis. The prediction remained unchanged in 373 patients, with 301 (70%) correct and 72 (17%) incorrect predictions (Table 3). Figure 3 shows correctly and incorrectly changed predictions in relation to the DSI values. Lower DSI values were associated with a higher number of patients with correctly changed predictions, whereas higher DSI values were associated with more incorrectly changed predictions.

**Table 3:** Impact of the PredictND tool on the baseline prediction of progression

With tool prediction (WT)	All (*n* = 429)		SCD (*n* = 230)		MCI (*n* = 199)	
According to FU diagnosis,	Correct	Incorrect	Correct	Incorrect	Correct	Incorrect
*n* (%)	330 (77)	99 (23)	197 (86)	33 (14)	133 (67)	66 (33)
Unchanged prediction, WOT = WO, *n* (%)	301 (70)	72 (17)	184 (80)	21 (9)	117 (59)	51 (26)
Changed prediction, WOT ≠ WO, *n* (%)	29 (7)	27 (6)	13 (6)	12 (5)	16 (8)	15 (7)
Confidence in VAS score (0–100%)						
Without tool confidence (WOT)	67 ± 15	60 ± 15	72 ± 15	61 ± 18	60 ± 12	60 ± 13
With tool confidence (WT)	72 ± 16	62 ± 17	79 ± 13	63 ± 19	62 ± 14	61 ± 15
Δ Difference confidence	5 ± 13*	2 ± 15	7 ± 10*	2 ± 17	2 ± 16	1 ± 14
Confidence (high/moderate/low)						
Increase in confidence (%)	83 (19)	23 (5)	54 (23)	9 (4)	29 (15)	14 (7)
Decrease in confidence (%)	25 (6)	13 (3)	8 (3)	6 (3)	17 (9)	7 (3)
Stable confidence (%)	222 (52)	63 (15)	135 (59)	18 (8)	87 (44)	45 (22)

The baseline predicted follow-up diagnosis with tool compared to the follow-up diagnosis for all patients and stratified according to baseline SCD and MCI diagnosis. “Unchanged prediction” indicates patients where the prediction did not change after the PredictND tool was used, whereas in “changed prediction,” the baseline predicted follow-up diagnosis without tool was changed when applying the tool. Data are presented as mean ± SD or *n* (%). Difference between without and with tool confidence was assessed using paired-sample *t* tests

*Abbreviations: WOT* without tool, *WT* with tool, *Δ difference confidence* the difference between confidence in the prediction without and with tool, *VAS* visual analogue scale from 0 to 100%

*Significant increased confidence after using the PredictND tool, *p* < 0.05

Table 4 presents the performance of clinicians without and with the PredictND tool, and the DSI classification alone. Across all patients, we found practically no difference in the prognostic accuracy between the clinician without (acc. = 76%) and with tool (acc. = 77%) (0.4%, 95%CI − 3.0%; + 3.9%, *p* = 0.79). When excluding patients who progressed to non-AD dementia, the difference in prognostic accuracies seemed to increase slightly (1.2%, 95%CI − 2.2%; + 4.7%; *p* = 0.48). Including only patients with DSI below 0.2 and above 0.8 (*n* = 203), the accuracy increased by 3.0% (95%CI − 0.6%; + 6.5%, *p* = 0.11) from 90 to 93% (Table 4). Overall, the PPVs were moderate, whereas the NPVs were high. A slightly higher NPV (0.96) and lower PPV (0.34 and 0.35) were observed for SCD compared to MCI (NPV = 0.78 and 0.81, and PPV = 0.48).

**Table 4:** Performance to predict progression for clinicians without and with the PredictND tool, and the DSI classification alone

Cohort	SN	SP	PPV	NPV	Accuracy	Bal. Acc.
All (*n* = 429)						
Without tool	0.67	0.79	0.43	0.91	0.76	0.73
With tool	0.60	0.81	0.43	0.89	0.77	0.70
DSI	0.63	0.83	0.47	0.90	0.79	0.73
Excl. non-AD dementia (*n* = 405)						
Without tool	0.67	0.79	0.35	0.93	0.77	0.73
With tool	0.62	0.81	0.36	0.92	0.78	0.71
DSI	0.63	0.83	0.39	0.93	0.80	0.73
SCD (*n* = 230)						
Without tool	0.67	0.87	0.34	0.96	0.85	0.77
With tool	0.67	0.88	0.35	0.96	0.86	0.77
DSI*	0.33	0.96	0.47	0.93	0.90	0.65
MCI (*n* = 199)						
Without tool	0.67	0.66	0.48	0.81	0.66	0.66
With tool	0.57	0.71	0.48	0.78	0.67	0.64
DSI	0.73	0.62	0.47	0.83	0.65	0.67
DSI ≤ 0.2 or DSI ≥ 0.8 (*n* = 203)						
Without tool	0.67	0.92	0.44	0.97	0.90	0.79
With tool	0.61	0.96	0.58	0.96	0.93	0.78
DSI	0.78	0.98	0.78	0.98	0.96*	0.88

*Abbreviations: Prog* conversion of SCD to MCI, AD or another type of dementia, and MCI to AD or another type of dementia; *SN* sensitivity; *SP* specificity; *PPV* positive predictive value; *NPV* negative predictive value; *Bal.Acc.* balanced accuracy; *DSI* disease state index; *SCD* subjective cognitive decline; *MCI* mild cognitive impairment

**Significant difference between without tool and DSI classification accuracy, *p* = 0.012

*Results from different cutoff values are available in Additional file 1: Table S8

The DSI classification alone performed at the same level as the clinicians. For the subgroups with DSI values below 0.2 or above 0.8, representing a more certain DSI classification for either progression or stability, the DSI classification alone had significant higher accuracy than the clinicians without tool (6.4%, 95%CI 2.1%; 10.7%; *p* = 0.004) (additional results in Additional file 1: Tables S2–S7).

### Confidence in the prediction of progression without and with the PredictND tool

In 34% (*n* = 144) of all patients, the level of confidence in the prediction of progression changed following the application of the PredictND tool. The confidence increased from low to moderate/high or from moderate to high in 19% (*n* = 83) of the correct and 5% (*n* = 23) of the incorrect predictions meaning that 78% of the 106 cases with increased confidence supported a correct prediction. The confidence decreased from high to moderate/low or from moderate to low in 6% (*n* = 25) of the correct and 3% (*n* = 13) of the incorrect predictions (Table 3).

Overall, confidence in the prediction increased significantly with 4% on the VAS scale (*p* < .0001) when applying the PredictND tool to the evaluation. For patients with correct predictions, the mean VAS score increased 5% (*p* < .0001) and for incorrect predictions the score increased by 2% (*p* = 0.33) (Table 3). The increase in the mean VAS score was highest for SCD patients with correct predictions (∆VAS = 7%, *p* < .0001). Furthermore, in patients with more extreme DSI values (DSI < 0.2 or DSI > 0.8), the clinician’s confidence in the predictions increased more (∆VAS = 7%, *p* < .0001) than in patients with medium DSI values (∆VAS = − 2%, *p* = 0.07) (*p* < .0001).

## Discussion

In this prospective multicenter study, we found that the prediction of progression in non-demented memory clinic patients was changed in 13% of all patients when the PredictND tool was applied. Particularly for patients with extreme DSI values (indicating either progression or stability with higher certainty), the use of the tool had a tendency to increase the prognostic accuracy and the DSI alone showed statistically significant improvement. Moreover, the clinicians’ confidence in the prediction increased when the PredictND tool was added to the evaluation, especially for patients with SCD.

In previous studies using retrospective data, we have shown that the DSI classifier can discriminate between stable and progressive MCI (acc. = 0.70–0.71), as well as SCD (balanced acc. = 0.74) [21–23, 25]. Moreover, DSI was able to identify half of the patients with MCI who progressed to a clinical AD diagnosis 12 months prior to the conversion with an accuracy of 88% [20]. The accuracy for MCI (acc. = 0.66–0.67) in the current multicenter study was similar to previous results in the retrospective studies, e.g., 0.65–0.75 in [37]. Furthermore, in this study, we extended the previous findings by applying the PredictND tool to a prospective mixed memory clinic cohort to evaluate the real-life impact of the tool.

Focusing on the patients with a higher certainty in the classification (DSI < 0.2 or > 0.8), which accounted for nearly half of the cohort, we found that use of the PredictND tool had a tendency to improve the prognostic accuracy (*p* = 0.11). However, when the tool was used alone in this patient group, a statistically significant difference was observed. This implies that clinicians could have trusted the tool more than they did when assessing the cases with extreme DSI values. The patients with DSI between 0.2 and 0.8 form an inconclusive group for which accurate prediction is not possible with given data. In previous studies, we have likewise found higher performance with more extreme DSI values, especially lower values (DSI < 0.2), emphasizing a clear strength of the tool for identifying patients who will remain stable [20, 21, 23, 25]. Our results showed high NPV, whereas the PPV was relatively low both for clinicians without and with tool, and for the DSI value alone [18, 25]. Therefore, when evaluating the whole cohort, the major value of the tool seems to be in establishing reassurance for patients who are unlikely to progress. However, for the extreme DSI values, the PPV was higher when the clinician was assisted by the tool mainly due to less false positive predictions. For this subgroup, the tool thus seems more precise in identifying the individuals at high risk of progression (risk ratios 5.0–8.8, see Additional file 1: Table S4) with a need for a closer follow-up within the clinically relevant period of 12–24 months.

High confidence in the prognosis is important to provide convincing reassurance to patients with a stable condition and to identify patients in need of *comprehensive* clinical follow-up or even early treatment. We found that the clinicians’ confidence in their prediction increased when using the PredictND tool. The highest increase in confidence was seen in correct predictions, especially for SCD patients. Moreover, as expected, the change in confidence was dependent on the DSI value with higher impact of the tool when the classification was more consistent.

The main strength of the study is the large well-characterized multicenter cohort making the result more generalizable. Moreover, the prospective design provided optimal conditions to test the tool in an actual clinical setting when a clinician has seen or obtained second-hand information of the patient.

Prospective studies are also associated with several potential limitations. First, the study design was a trade-off between the importance of retaining the clinician’s impression of the patient and minimizing bias carried over from the first to the second evaluation. Thus, in some cases, the time between the evaluations was longer than intended and it might have affected the result. Furthermore, to evaluate progression in patients with MCI and especially SCD, the follow-up time was short [1, 5, 18]. Though, for clinical use particularly in patients with MCI, it may be more relevant to identify the impact on prediction of progression within 1–2 years rather than distant future. Generally, the number of progressors was low, but it corresponded to estimated annual conversion rates in other studies [1, 5]. In a few patients, the clinical condition improved, and these cases were handled as stable in the analysis as the tool’s impact in terms of reassurance was considered similar. Relatively broad entry criteria may have also led to inclusion of patients with mild dementia rather than strictly MCI. However, baseline characteristics (e.g., MMSE and CDR) indicate that this was seldomly the case. Another issue is the use of training data from controls and AD patients. However, our previous studies demonstrate that the classification performance is comparable independent on whether the model is trained with data from SCD and AD patients or from stable and progressive MCI patients [19, 20, 25]. Yet, when the clinician interprets the DSI values, the choice of AD and controls as training data might be associated with some considerations. For MCI patients, the DSI values are closer to the cutoff value (DSI = 0.5) than if the model was trained using stable and progressive MCI subjects [19, 25]. For SCD patients considering progression to MCI, the cutoff value (DSI = 0.5) is not optimal as many MCI patients will have a DSI value lower than 0.5. This explains why sensitivity was low (0.33) and specificity was high (0.96) in the SCD group using DSI (performance at different cutoff values for SCD is presented in Additional file 1: Table S8). In this study, the cutoff was not optimized separately for SCD and MCI patients but the same cutoff (DSI = 0.5) was used for all patients. Refinement of the prediction algorithm accounting for, e.g., different cutoff values may improve the results. Finally, specific models for non-AD dementias were not applied and our analysis excluding patients who progressed to non-AD dementia showed a slight increase in the prognostic accuracy (see Additional file 1: Table S2), suggesting that training data with cohorts of non-AD dementias may improve the clinical importance of the tool.

Various studies with cognitive tests, CSF, and MRI biomarkers have shown the prognostic value of diagnostic tests on group levels, whereas predictive classifiers have the advantage of providing predictions on an individual patient level with MCI and SCD [2, 3, 9–13]. In additional, the PredictND tool, based on the DSI classifier, also analyzes large quantities of heterogeneous patient data and enables the clinician to easily interpret the results visually [19, 26]. Other commonly used classifiers are logistic regression models, Bayesian classifiers, support vector machine (SVM), and random forest [19, 41–44]. Another promising prognostic model interpreting MRI and CSF in the light of age, gender, and MMSE to provide a progression risk has recently been introduced for clinical use, but to our knowledge, the clinical impact has not yet been investigated [18]. These and other emerging prognostic models represent potentially very valuable clinical support tools for the clinicians in the future. However, to ensure optimal quality for the patients, it is important to evaluate and compare the performance and clinical impact, and preferably in a standardized manner [17].

## Conclusions

Findings from this study indicate that the PredictND tool affected the prediction of progression for SCD and MCI both in terms of changing the clinicians’ predictions and increasing their confidence. Although no statistically significant difference was observed when using the tool, the results show potential for improvements especially for patients with most extreme DSI values (DSI classifications < 0.2 or > 0.8). The tool alone showed an increase in accuracy (statistically significant) compared with the situation when no tool was used in the patients with DSI < 0.2 or DSI > 0.8. In this subpopulation, stable patients were identified with high accuracy. Furthermore, our results indicate that decision support tools in the future could make clinicians more confident in their short-term prognosis by providing a decent second opinion in prognostic decision-making.

## Additional files

Additional file 1: Presents additional results, such as demographics, accuracy, and performance for subgroups. (PDF 2187 kb)

## Abbreviations

18F-FDG-PET: 18F-fluorodeoxyglucose positron emissions tomography
AD: Alzheimer’s disease
Aβ42: Amyloid-β 1–42
CDR: Clinical dementia rating
CSF: Cerebrospinal fluid
DSF: Disease state fingerprint
DSI: Disease state index
FLAIR: Fluid-attenuated inversion recovery
MCI: Mild cognitive impairment
MRI: Magnetic resonance imaging
MTA: Medial temporal lobe atrophy
NPV: Negative predictive value
PPV: Positive predictive value
ROI: Region-of-interest
SCD: Subjective cognitive decline
VAS: Visual analogue scale
WMH: White matter hyperintensities
