# Supplementary material for: Impact of a clinical decision support tool on prediction of progression in early-stage dementia: a prospective validation study
Source: Alzheimers Res Ther. 2019 Mar 20;11:25. doi: 10.1186/s13195-019-0482-3 (PMC6425602; doi:10.1186/s13195-019-0482-3)
Supplement: Supplementary file 1 — Presents additional results, such as demographics, accuracy, and performance for subgroups. (PDF 2187 kb) [file 13195_2019_482_MOESM1_ESM.pdf]

ADDITIONAL FILE 1

Table S1. Baseline characteristics according to outcome at follow-up

Characteristic	n	SCD		MCI		Significant differences between groups
		**Stable n = 209**	**Progressed n = 21**	**Stable n = 136**	**Progressed n = 63**	
Demographics						
Female, n (%)	429	140 (67)	10 (48)	51 (38)	29 (46)	All<sSCD
Age, years	429	63 ±9	71 ±8	69 ±9	73 ±8	sSCD<All, sMCI<pMCI
Duration of symptoms, years	380	3 ±4	4 ±4	2 ±3	2 ±2	-
MCI/AD/non-AD, n		-	16/3/2	-	0/41/22	NA
CDR, n (0.0/0.5/1.0)	424	170/37/1	12/9/0	28/99/5	11/48/4	NA
APOE status						
APOE e4 carrier, n (%)	146	30 (14)	5 (24)	20 (15)	9 (14)	-
Cognitive tests						
MMSE	427	29 ±1	28 ±2	27 ±2	26 ±3	pMCI<All, sMCI<sSCD, pSCD
Memory – Learning	420	45 ±10	32 ±9	37 ±11	31 ±10	All<sSCD, pMCI<sMCI
Memory – Recall	420	10 ±3	6 ±2	7 ±4	4 ±3	pMCI<sMCI, pSCD<sSCD
TMT-A, seconds	422	37 ±17	45 ±15	49 ±21	62 ±32	All<pMCI, sSCD<sMCI
TMT-B, seconds	402	81 ±39	128 ±57	138 ±71	177 ±86	sSCD<pSCD, sMCI<pMCI
Fluency – animal	407	25 ±7	21 ±4	20 ±6	17 ±6	pMCI<sMCI, pSCD<sSCD
Fluency – Letter	377	15 ±5	13 ±5	12 ±5	11 ±5	pMCI, sMCI<sSCD,
Clock-drawing	394	3 ±0	3 ±1	3 ±1	2 ±1	pMCI<sMCI<sSCD,
CSF						
Aβ42, pg/ml	145	971 ±264	761 ±323	894 ±302	743 ±350	pMCI<sSCD
P-tau, pg/ml	145	50 ±21	84 ±49	55 ±27	55 ±21	All<pSCD
Total tau, pg/ml	145	325 ±173	681 ±507	371 ±218	360 ±158	All<pSCD
MRI – visual scores						
GCA (median, Q1-Q3)	418	0.4 ±0.6 (0, 0-1)	1.0 ±0.8 (1, 0-1)	1.0 ±0.8 (1, 0-1)	1.3 ±0.8 (1, 1-2)	sSCD<All, pSCD, sMCI<pMCI
MTA, right (median, Q1-Q3)	398	0.4 ±0.6 (0, 0-1)	0.9 ±0.8 (1, 0-1)	1.0 ±0.9 (1, 0-1)	1.6 ±1.0 (1, 1-2)	sSCD<pSCD, sMCI<pMCI
MTA, left (median, Q1-Q3)	398	0.3 ±0.6 (0, 0-1)	1.0 ±0.8 (1, 1-1)	0.9 ±0.9 (1, 0-1)	1.7 ±1.0 (2, 1-2)	sSCD<pSCD, sMCI<pMCI
Fazekas score (median, Q1-Q3)	420	0.7 ±0.7 (1, 0-1)	1.0 ±0.7 (1, 1-2)	1.1 ±0.8 (1, 1-2)	1.1 ±0.9 (1, 1-2)	sSCD<pMCI, sMCI

Abbreviations: SCD: Subjective cognitive decline, MCI: Mild cognitive impairment, CDR: clinical dementia rating (global score, range 0-3), MMSE: the Mini-Mental State Examination, Memory: the Rey Auditory Verbal Learning Test (RAVLT) values, using z-scoring for those with only the Consortium to Establish a Registry for Alzheimer's Disease (CERAD) word list memory test, TMT: the Trail Making Test, CSF: Cerebrospinal fluid, Aβ42: Amyloid beta 1-42, p-tau: tau phosphorylated at threonine 181, MRI: Magnetic resonance imaging, GCA: global cortical atrophy, MTA: medial temporal lobe atrophy, sSCD: stable SCD, pSCD: progressed SCD, sMCI: stable MCI, pMCI: progressed MCI.

Note: Differences between groups were assessed using one-way ANOVA with post-hoc Bonferroni tests, $\chi2$ tests and Kruskal–Wallis with post-hoc Wilcoxon rank-sum test. Data are presented as mean ± SD unless otherwise specified. Significant difference, $p<0.05$.

*history of depression (medical treatment or hospitalization)

**Diabetes, Myocardial Infarction, Atrial Fibrillation, Previous Stroke, Arterial Hypertension or Hypercholesterolemia

Table S2. Difference in prognostic accuracy and confidence between without vs. with tool evaluation, stratified by different subgroups

	All					DSI≤0.2 or DSI≥0.8				
Cohort	n	Accuracy	p-value	Mean VAS	p-value	n	Accuracy	p-value	Mean VAS	p-value
All	429	0.4 (-3.0;3.9)	0.79	4.1 (13)	<.0001	203	3.0 (-0.6;6.5)	0.11	6.7 (11)	<.0001
Excl. non-AD	405	1.2 (-2.2;4.7)	0.48	4.2 (13)	<.0001	199	3.5 (0.0;7.0)	0.05	6.8 (11)	<.0001
SCD	230	0.4 (-3.8;4.7)	0.84	6.0 (11)	<.0001	162	3.7 (0.0;7.5)	0.06	6.9 (10)	<.0001
MCI	199	0.5 (-5.0;6.0)	0.86	2.0 (15)	0.03	41	0.0 (-9.6;9.6)	1.0	6.0 (13)	0.006
Available biomarkers										
MRI+NPS+CSF	145	4.1 (-2.4;10.7)	0.22	2.8 (16)	0.04	44	-2.3 (-12.2;7.7)	0.65	5.4 (15)	0.019
MRI+NPS, excl. CSF	283	-1.4 (-5.3;2.5)	0.48	4.8 (12)	<.0001	159	4.4 (0.8;8.0)	0.018	7.1 (10)	<.0001
MRI+NPS, excl. CSF+APOE	246	-1.6 (-5.9;2.6)	0.45	5.5 (11)	<.0001	140	5.0 (0.9;9.1)	0.017	7.9 (9)	<.0001
MRI+NPS, Excl. all biomarkers*	205	-1.5 (-6.3;3.3)	0.55	6.2 (9)	<.0001	123	4.9 (0.5;9.3)	0.031	8.3 (9)	<.0001

Abbreviations: DSI: The disease state index, VAS: visual analogue scale from 0-100%, Excl. non-AD: Excluding all non-AD dementia patients, SCD: Subjective cognitive decline, MCI: Mild cognitive impairment, All biomarkers: CSF, MRI, *APOE*, EEG, Genetic testing, DAT SPECT, FDG-PET or amyloid PET.

Table S3. Impact of the PredictND tool on the baseline prediction of progression, non-AD dementia excluded

**With tool predicted diagnosis (WT)**	**All cases (n=405)**		**SCD (n=228)**		**MCI (n=177)**	
According to FU diagnosis	Correct	Incorrect	Correct	Incorrect	Correct	Incorrect
n	317 (78)	88 (22)	195 (86)	33 (14)	122 (69)	55 (31)
Unchanged prediction, WOT=WO, n (%)	289 (71)	65 (16)	182 (80)	21 (9)	107 (61)	44 (25)
Changed prediction, WOT≠WO, n (%)	28 (7)	23 (6)	13 (6)	12 (5)	15 (8)	11 (6)
**Confidence in VAS score (0-100%)**						
Without tool confidence (WOT)	68 ±15	60 ±15	72 ±15	61 ±18	60 ±12	60 ±14
With tool confidence (WT)	73 ±16	62 ±17	79 ±13	63 ±19	62 ±14	62 ±15
Δ Difference confidence	5 ±13*	2 ±15	7 ±10*	2 ±17	2 ±16	2 ±13
**Confidence (High/Moderate/Low)**						
Increase in confidence (%)	78 (19)	22 (5)	53 (23)	9 (4)	25 (14)	13 (7)
Decrease in confidence (%)	24 (6)	12 (3)	8 (4)	6 (2)	16 (9)	6 (3)
Stable confidence (%)	215 (53)	54 (14)	134 (59)	18 (8)	81 (46)	36 (21)

Abbreviations: WOT: Without tool, WT: With tool, Δ Diagnostic confidence: the difference between confidence in the prediction without and with tool, VAS: visual analogue scale from 0-100%.
Note: The baseline prediction of progression without versus with tool compared to the follow-up diagnosis for all patients and stratified according to baseline SCD and MCI diagnosis. “Unchanged prediction” indicates patients where the prediction did not change after the PredictND tool was used, whereas in “Changed prediction” the baseline predicted follow-up diagnosis without tool was changed when applying the tool. Data are presented as mean ± SD or n (%). Difference between without and with tool confidence was assessed using paired-sample *t* tests.
*Significant increased prognostic confidence after using the PredictND tool, $p < 0.05$

Table S4. Performance to predict progression for clinicians without and with the PredictND tool, and the DSI classification alone – extended version

	N	Prog	Stable	SN	SP	PPV	NPV	Acc.	Bal. Acc.	AUC	Risk-ratio**
**All, n (prog/stable)**	429	(n=84)	(n=345)								
Without tool (129/300)		56/28	73/272	0.67	0.79	0.43	0.91	0.76	0.73		2.2
With tool (115/314)		50/34	65/280	0.60	0.81	0.43	0.89	0.77	0.70		2.2
DSI (113/316)		53/31	60/285	0.63	0.83	0.47	0.90	0.79	0.73	0.82	2.4
**SCD**	230	(n=21)	(n=209)								
Without tool (41/189)		14/7	27/182	0.67	0.87	0.34	0.96	0.85	0.77		3.7
With tool (40/190)		14/7	26/183	0.67	0.88	0.35	0.96	0.86	0.77		3.8
DSI (15/215)		7/14	8/201	0.33	0.96	0.47	0.93	0.90	0.65	0.84	5.1
**MCI**	199	(n=63)	(n=136)								
Without tool (93/119)		42/21	46/90	0.67	0.66	0.48	0.81	0.66	0.66		1.5
With tool (75/124)		36/27	39/97	0.57	0.71	0.48	0.78	0.67	0.64		1.5
DSI (98/101)		46/17	52/84	0.73	0.62	0.47	0.83	0.65	0.67	0.73	1.5
**DSI≤0.2 or DSI≥0.8**	203	(n=18)	(n=185)								
Without tool (27/176)		12/6	15/170	0.67	0.92	0.44	0.97	0.90	0.79		5.0
With tool (19/184)		11/7	8/177	0.61	0.96	0.58	0.96	0.93	0.78		6.5
DSI (18/185)		14/4	4/181	0.78	0.98	0.78	0.98	0.96 [a]	0.88	0.92*	8.8

Abbreviations: Prog: conversion of SCD to MCI, AD or another type of dementia, and MCI to AD or another type of dementia, SN: sensitivity, SP: specificity, PPV: positive predictive value, NPV: negative predictive value, Acc.: accuracy, Bal.Acc.: balanced accuracy, DSI: disease state index, SCD: subjective cognitive decline, MCI: mild cognitive impairment.
* Should be interpreted with caution as the Disease State Index (DSI) not just is a dichotomic classifier but also gives information for each patient on how reliable the classification is. More precise AUC are presented in Rhodius-meester HFM et al., Computer-assisted prediction of clinical progression in the earliest stages of AD. Alzheimer's & Dementia: Diagnosis, Assessment & Disease Monitoring, 2018; Oct.. pp 1-11.
** Risk-ratio was calculated as probability of progression in the subpopulation (e.g. PPV=0.43 for "All Without tool") divided by probability of progression in the whole population (e.g., 84/(84+345) for "All Without tool") giving risk ratio 2.2.
[a] Significant difference between without tool and DSI classification accuracy, P=0.012

Table S5. Performance to predict progression for clinicians without and with the PredictND tool, and the DSI classification alone – non-AD dementia excluded

	N	Prog	Stable	SN	SP	PPV	NPV	Acc.	Bal. Acc.
**All, n (prog/stable)**	405	(n=60)	(n=345)						
Without tool (113/292)		40/20	73/272	0.67	0.79	0.35	0.93	0.77	0.73
With tool (102/303)		37/23	65/280	0.62	0.81	0.36	0.92	0.78	0.71
DSI (98/307)		38/22	60/285	0.63	0.83	0.39	0.93	0.80	0.73
**SCD**	228	(n=19)	(n=209)						
Without tool (39/189)		12/7	27/182	0.63	0.87	0.31	0.96	0.85	0.75
With tool (38/190)		12/7	26/183	0.63	0.88	0.32	0.96	0.86	0.75
DSI (14/214)		6/13	8/201	0.32	0.96	0.43	0.94	0.91	0.64
**MCI**	177	(n=41)	(n=136)						
Without tool (74/103)		28/13	46/90	0.68	0.66	0.38	0.87	0.67	0.67
With tool (64/113)		25/16	39/97	0.61	0.71	0.39	0.86	0.69	0.66
DSI (84/93)		32/9	52/84	0.78	0.62	0.38	0.90	0.66	0.70
**DSI≤0.2 or DSI≥0.8**	199	(n=14)	(n=185)						
Without tool (25/174)		10/4	15/170	0.71	0.92	0.40	0.98	0.90	0.82
With tool (18/181)		10/4	8/177	0.71	0.96	0.56	0.98	0.94	0.84
DSI (14/185)		10/4	4/181	0.71	0.98	0.71	0.98	0.96	0.85

Abbreviations: Prog: conversion of SCD to MCI, AD or another type of dementia, and MCI to AD or another type of dementia, SN: sensitivity, SP: specificity, PPV: positive predictive value, NPV: negative predictive value, Acc.: accuracy, Bal.Acc.: balanced accuracy, DSI: disease state index, SCD: subjective cognitive decline, MCI: mild cognitive impairment.

Table S6. Performance at different DSI cutoff values

Cohort	n	Percentage	SN	SP	Bal.Acc.
Cut-off 0.5	429	100	0.63	0.83	0.73
DSI<0.4 or DSI>0.6	358	83	0.61	0.87	0.74
DSI<0.3 or DSI>0.7	280	65	0.60	0.93	0.77
DSI<0.2 or DSI>0.8	203	47	0.78	0.98	0.88
DSI<0.1 or DSI>0.9	116	27	0.83	0.97	0.90

Abbreviations: SN: sensitivity, SP: specificity, PPV: positive predictive value, NPV: negative predictive value, Acc.: accuracy, Bal.Acc.: balanced accuracy

Table S7. Correspondence between without and with tool prediction, the DSI classification and the follow-up reference status, All cases

	**All**		**SCD**		**MCI**	
	**FU (stable)**	**FU (prog)**	**FU (stable)**	**FU (prog)**	**FU (stable)**	**FU (prog)**
**WOT/WT (stable/stable)**						
DSI (stable)	224 (52)	12 (3)	164 (73)	4 (2)	56 (28)	8 (4)
DSI (prog)	31 (7)	12 (3)	5 (2)	0 (0)	26 (13)	12 (6)
**WOT/WT (stable/prog)**						
DSI (stable)	12 (3)	2 (1)	9 (4)	1 (1)	3 (1)	1 (1)
DSI (prog)	5 (1)	2 (1)	0 (0)	2 (1)	5 (2)	0 (0)
**WOT/WT (prog/prog)**						
DSI (stable)	27 (6)	14 (3)	14 (6)	7 (3)	13 (7)	7 (4)
DSI (prog)	21 (5)	32 (7)	3 (1)	4 (2)	18 (9)	28 (14)
**WOT/WT (prog/stable)**						
DSI (stable)	24 (6)	6 (1)	10 (4)	3 (1)	14 (7)	3 (1)
DSI (prog)	1 (0)	4 (1)	0 (0)	0 (0)	1 (1)	4 (2)

Abbreviations: SCD: subjective cognitive decline, MCI: mild cognitive impairment, FU: follow-up status. prog: conversion of SCD to MCI, AD or another type of dementia, and MCI to AD or another type of dementia, WOT: without the PredictND tool, WT: with the PredictND tool.
The correspondence between without and with tool prediction, the DSI classification and the follow-up status (WOT/WT/DSI/FU) in regard to whether the syndrome diagnosis remained stable or progressed (stable/prog), for all patients and stratified according to baseline SCD and MCI diagnosis. Data are presented as n (%).

Table S8. Performance of DSI classification at different cut-off values for SCD

**Cohort**	**SN**	**SP**	**PPV**	**NPV**	**Accuracy**	**Bal.Acc.**
Cut-off 0.5	0.33	0.96	0.47	0.93	0.90	0.65
Cut-off 0.45	0.43	0.95	0.47	0.94	0.90	0.69
Cut-off 0.4	0.43	0.92	0.36	0.94	0.88	0.68
Cut-off 0.35	0.52	0.91	0.37	0.95	0.87	0.72
Cut-off 0.3	0.62	0.84	0.28	0.96	0.81	0.73
Cut-off 0.25	0.71	0.78	0.25	0.96	0.77	0.75
Cut-off 0.2	0.81	0.73	0.23	0.97	0.74	0.77
Cut-off 0.15	0.86	0.59	0.17	0.98	0.61	0.72
Cut-off 0.1	1.0	0.41	0.14	1.0	0.46	0.70

Abbreviations: SN: sensitivity, SP: specificity, PPV: positive predictive value, NPV: negative predictive value, Acc.: accuracy, Bal.Acc.: balanced accuracy
